# *Zebrafish* as a Model Organism in Space Biology Research

**DOI:** 10.3390/biology15211983

**Published:** 2026-11-02

**Authors:** Tianyi Er, Chen Zhang, Ziming Wang, Sisi Ruan, Jian Mao, Hong Ma

**Affiliations:** 1 Flavor Science Laboratory, Beijing Life Science Academy (BLSA), Beijing 102209, China; 2 Key Laboratory of Biosynthesis and Biomanufacturing in Model Plants (Beijing Life Science Academy), Ministry of Industry and Information Technology, Beijing 102209, China; 3 School of Medical Technology, Beijing Institute of Technology, Beijing 100081, China

**Keywords:** *zebrafish*, space environment, microgravity, radiation, physiological damage, molecular mechanisms

## Abstract

As deep-space exploration advances, the health effects of the space environment have become a critical concern. *Zebrafish* (*Danio rerio*) have emerged as a valuable vertebrate model for space biology owing to their genetic tractability, optical transparency, and conserved organ systems. This review summarizes recent advances in using *zebrafish* to study spaceflight-associated health risks, focusing on ionizing radiation and altered gravity. We examine effects across the cardiovascular, nervous, musculoskeletal, sensory, reproductive, and other systems. Radiation induces cardiac dysfunction, neurobehavioral deficits, skeletal deformation, ocular damage, and reproductive toxicity, often through oxidative stress, apoptosis, and dysregulated signaling pathways. Microgravity and hypergravity primarily affect the musculoskeletal and vestibular systems, with microgravity reducing bone ossification and impairing otolith development. We also discuss current limitations, including the gap between ground-based simulations and actual space conditions, and the lack of combined-stressor studies. Finally, we highlight future directions, such as in-orbit live imaging, CRISPR/Cas9-based validation, and high-throughput countermeasure screening. This review aims to provide a theoretical framework for using *zebrafish* to assess space-environment-associated health risks and to inform research on health risks relevant to long-duration spaceflight.

## Simple Summary

Spaceflight can harm astronauts’ hearts, brains, muscles, bones, eyes, and reproductive systems. To study these risks, scientists need animal models that are easy to observe and share similar biology with humans. *Zebrafish* are small, transparent, breed quickly, and have many organs that work like ours. This review summarizes how *zebrafish* have been used to study space conditions, especially radiation and changes in gravity. Radiation mainly damages the heart, brain, muscles, eyes, and reproductive system, while altered gravity affects bones, muscles, and sensory system. *Zebrafish* studies have helped explore mechanisms of injury and identify candidate protective compounds. Although ground-based simulations cannot fully reproduce spaceflight conditions, *zebrafish* remain a complementary model for informing astronaut health research.

## 1. Introduction

In 1961, Soviet cosmonaut Yuri Gagarin completed the first human orbital flight aboard Vostok-1, marking the beginning of human space exploration. As deep-space exploration expands, concerns regarding cumulative health risks have intensified [1,2]. The landmark Twin Study conducted by the National Aeronautics and Space Administration (NASA) systematically demonstrated that prolonged exposure to the space environment induces multi-system alterations, including epigenetic reprogramming, cardiovascular deconditioning, and cognitive impairment [3]. Consequently, ensuring astronaut health during long-duration spaceflight and elucidating the mechanisms of spaceflight-induced physiological damage have emerged as key priorities in space life sciences research.

The space environment differs substantially from Earth in multiple aspects, including ionizing radiation, microgravity, confinement, and disrupted circadian rhythms. Among these factors, space radiation and microgravity are recognized as two principal risk factors [4]. The most common types of space radiation include solar particle events (SPE) and galactic cosmic rays (GCR), along with secondary particles produced by the interactions of primary particles (SPE/GCR) with shielding materials. These radiations are categorized as high linear energy transfer (LET) and low-LET, which induce distinct patterns of damage to biomolecules and cellular structures [5]. During orbital flight and the cruise phase of an interplanetary mission, spacecraft and their occupants remain in near-continuous free fall and therefore experience microgravity. Microgravity, defined as a gravitational level on the order of 10^−4^ to 10^−6^ of Earth gravity, alters fluid dynamics and mechanical signaling, thereby influencing cellular behavior and tissue homeostasis [6,7]. In contrast, hypergravity refers to conditions in which effective acceleration exceeds normal gravity at Earth’s surface. During spaceflight, it occurs mainly for brief periods during launch and atmospheric re-entry [8].

Since direct spaceflight experiments are difficult to perform, ground-based simulation of the space environment is essential. Ground-based radiation studies commonly use accelerator-generated high-energy proton or heavy-ion beams, whereas low-LET radiation, such as γ-rays or X-rays, is often used as a simplified reference exposure for mechanistic investigations [9,10,11]. Compared with simulated hypergravity, simulated microgravity (SMG) has been more extensively investigated. For cells, tissues, embryos, and small organisms, SMG is commonly modeled using rotary culture systems, random positioning machines, and magnetic or acoustic levitation [12,13]. Notably, parabolic flights can provide brief periods of actual microgravity [14]. When increased gravity is investigated, centrifugation can be used to generate hypergravity in cell cultures and animal models [8]. However, some terrestrial radiation studies often involve short-term, high-dose or high-dose-rate exposure, whereas spaceflight involves prolonged, low-dose-rate exposure to a complex mixed radiation field [15]. Moreover, interactions among radiation, altered gravity, and other spaceflight factors may not be captured separately. Therefore, biological responses to ground-based simulations cannot fully represent those expected during actual spaceflight.

As early as 1947–1952, the U.S. V-2 and Aerobee rocket series successfully investigated key physiological parameters and neural vestibular function changes in various animal models during suborbital flights. Subsequently, Sputnik 2 carried the first animal into Earth orbit, and Sputnik 5 achieved the first safe return of animals from orbit [16]. In recent decades, in-orbit biological experiments have continued to evolve. The China Space Station’s Closed Aquatic Ecosystem (CSS-CAES) supported a *zebrafish* experiment in 2024, followed by the implementation of a mouse study in 2025 [17]. Among available model organisms, *zebrafish* have emerged as a key system compatible with both mechanistic research and orbital validation, owing to their high genetic and physiological conservation with humans, transparent embryos, and short reproductive cycle [3]. Ground-based simulations of microgravity and radiation using *zebrafish* have produced extensive data on skeletal metabolism, muscle atrophy, neural development, and cardiovascular function, providing a solid basis for their structured application in space health risk assessment.

This narrative review examines recent advances in *zebrafish* research under simulated space conditions. It aims to elucidate the underlying physiological and molecular mechanisms, to assess potential health risks in deep space exploration, and to provide insights for future space life science studies and astronaut health protection strategies.

## 2. *Zebrafish* as a Versatile Model Organism

**Figure 1:**
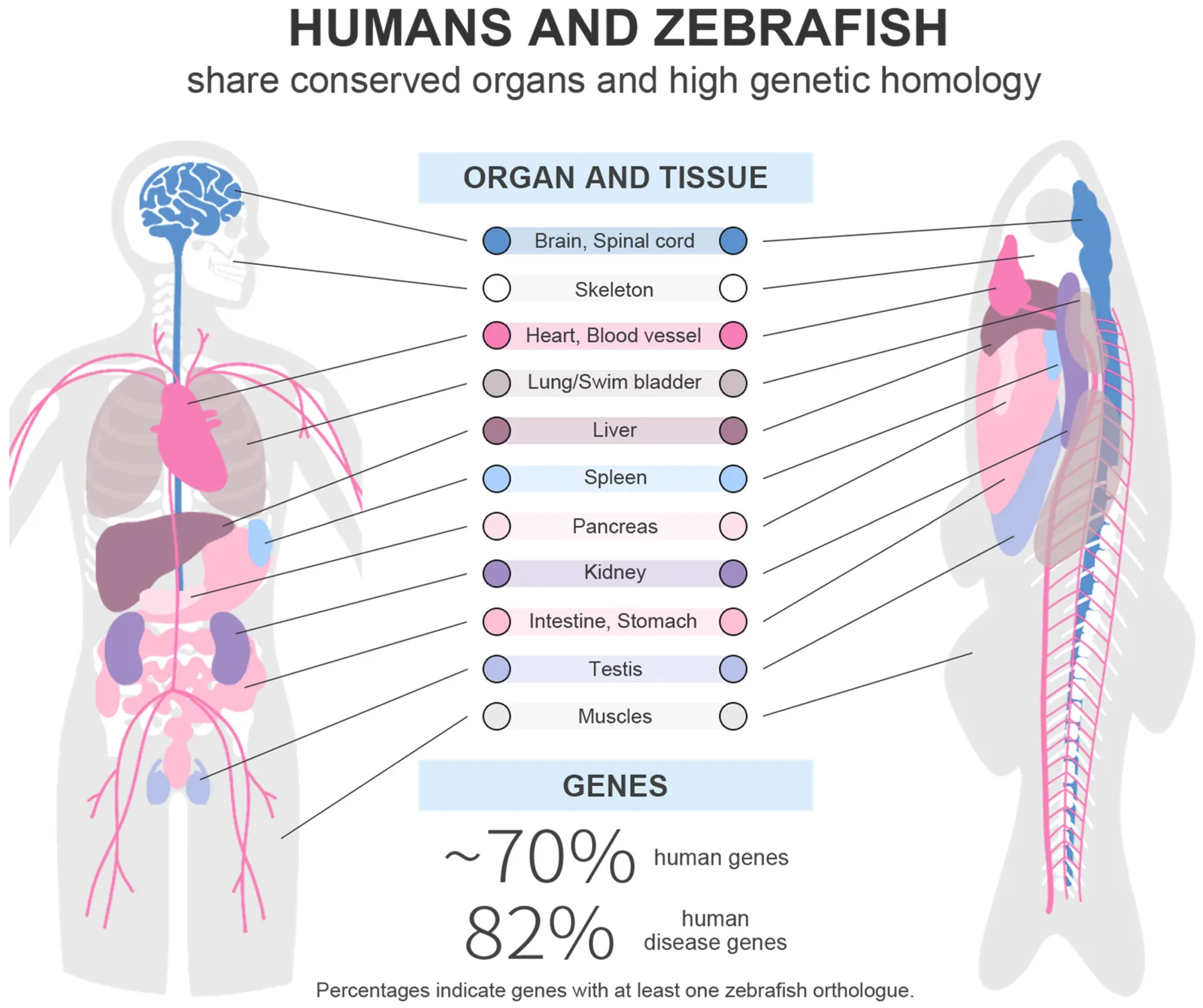
*Zebrafish* share conserved organs and high genetic homology with humans.

Since George Streisinger introduced *zebrafish* embryos into developmental research in the 1980s, this model organism has become an essential system for life science research [18]. Comparison to the human reference genome shows that approximately 70% of human genes have at least one obvious *zebrafish* orthologue [19]. As shown in Figure 1, its major organ systems, including cardiovascular, nervous, musculoskeletal, and other systems, are largely conserved with those of humans, positioning it as an ideal model for studying physiological functions [20]. Compared to mammalian models, *zebrafish* offer distinct advantages, including small size, high reproductive capacity, and external embryonic development, which facilitates experimental manipulation [21,22].

*Zebrafish* exhibit rapid early development, with embryos becoming suitable for experimental use as larvae by 3 days post-fertilization (dpf) and reaching sexual maturity by 90 dpf [23]. *Zebrafish* eggs are relatively large compared to other fish species (~0.7 mm in diameter at fertilization), and the optical clarity of embryos and larvae enables real-time, non-invasive live imaging. Developmental and functional abnormalities, including pericardial edema, venous thrombosis, spinal curvature, and tail malformation, are directly observable by microscopy [24,25]. When combined with fluorescent labeling techniques, *zebrafish* also enable real-time tracking of cellular development and apoptosis [26]. Furthermore, *zebrafish* require lower maintenance costs and less space and have an extended lifespan (3–5 years), making them an economical and efficient model organism. In disease research, 2601/3176 (82%) of human disease-associated genes have at least one *zebrafish* orthologue [19,27]. According to large-scale forward genetic screens, *zebrafish* mutants cover nearly all organ systems and cell types identified, most of which exhibit high conservation with mammals [19,28]. Currently, *zebrafish* have demonstrated significant potential in diverse fields, including cancer biology and in vivo drug screening, highlighting their established value in elucidating disease mechanisms [29,30].

In space biology research, long-term orbital studies utilizing *zebrafish* and other fish species have already established a solid foundation. The Skylab 3 mission in 1973 marked the first instance of sending mummichogs (*Fundulus heteroclitus*) into space within a polyethylene bag aquarium. In 1998, the Closed Equilibrated Biological Aquatic System (CEBAS) was developed and successfully sustained swordtail fish for 16 days, further demonstrating the broad feasibility of long-term aquatic experiments in space [31,32]. Recently, CSS-CAES has represented a major advancement in experimental duration, cultivation capacity, and the number of organisms. During the 43-day mission, *zebrafish* completed growth, development, and reproduction in this closed aquatic ecosystem, demonstrating the feasibility of long-duration vertebrate aquatic experiments in space. It maintains gas cycling through hornwort photosynthesis and *zebrafish* respiration, while intelligent LED lighting and liquid pumps optimize dissolved oxygen concentration and enhance material exchange [17].This breakthrough provides an essential platform and technical foundation for vertebrate biological research in space.

## 3. Effects on Major Physiological Systems

To facilitate comparison across the available evidence, the primary studies discussed in this section are summarized in Table 1. The table includes the *zebrafish* model and developmental stage, exposure conditions, and major physiological outcomes. The following subsections discuss these findings according to the major physiological systems.

### 3.1. Cardiovascular System

Spaceflight could induce acute cardiovascular alterations in astronauts, including post-flight orthostatic intolerance with impaired heart rate variability and baroreflex function, as well as in-flight arrhythmias such as ventricular ectopy [33]. In addition, exposure to cosmic radiation during space travel might induce late cardiovascular disease and accelerate atherosclerosis [33,34]. Given their unique physiological advantages, *zebrafish* offer an ideal model for studying cardiovascular diseases and spaceflight-induced cardiotoxicity [35].

Heart rate can be easily measured in the early *zebrafish* embryo and serves as a common indicator of radiation-induced cardiotoxicity [36]. For example, studies have reported decreased heart rate in embryos exposed to high-LET radiation, such as ^56^Fe ion and ^12^C ion [36,37,38]. Low-LET radiation can also affect the heart rate of *zebrafish* embryos [39]. Low-dose (1 and 2 Gy) and very low-dose (0.01, 0.05, and 0.1 Gy) γ-irradiation sightly increased in heart rate, while high-dose (10 Gy) γ-irradiation caused a significant decrease in heart rate in *zebrafish* embryo [40,41]. In addition to functional impairment, radiation also induces overt structural damage, with pericardial edema recognized as a classic morphological hallmark of severe toxicity, particularly at high doses of both high- and low-LET radiation [36,37,39,42]. Pericardial edema is usually accompanied by a decreased heart rate in *zebrafish* embryos with high-dose radiation treatment. Further anatomical observations by Xu et al. revealed that γ-irradiation induced cardiac enlargement and abnormal atrioventricular structure, which could be ameliorated by UA312 (optimized derivative of Ursolic acid) [43]. Although oxidative stress and apoptosis have been proposed as the primary contributing factors of radiation-induced cardiotoxicity, current insights are primarily derived from whole-embryo rather than cardiac-specific investigations [36,37,39,44]. As a result, the precise mechanisms underlying cardiac susceptibility remain to be fully elucidated.

Radiation also affected vascular formation and function. Jang et al. used a flk-1-GFP transgenic *zebrafish*, which contained fluorescent endothelial cells in the vasculature, as a module to explore the effect of a proton beam on blood vessel formation in *zebrafish* embryos. They found that a proton beam inhibited the development of vessels, and the antioxidant NAC reversed this effect [45]. Vala et al. reported that 0.5 Gy X-ray irradiation accelerated subintestinal-vessel sprouting in *zebrafish* exposed at 3 dpf and increased inter-ray vessel density during caudal-fin regeneration in adults [46]. Together, these findings raise the possibility of a biphasic vascular response to radiation. However, differences in experimental conditions and measured endpoints mean that a threshold effect cannot be excluded. Controlled dose–response studies are needed to test this hypothesis. Additionally, Xu et al. reported that γ-irradiation significantly increased venous thrombus formation in the tails of *zebrafish* larvae, characterized by the accumulation of red blood cells [43]. Taken together, these studies indicated that radiation can lead to abnormal development of the vascular system. Excessive angiogenesis may enhance the formation of microvessels, thereby increasing the risk of tumor growth and metastasis [47,48]. In adult *zebrafish*, Marques et al. further showed that low-dose ionizing radiation enhanced angiogenic responses during caudal-fin regeneration, as indicated by increased inter-ray vessel density. However, radiation did not accelerate overall fin regeneration or alter the expression of early regeneration markers, suggesting that its effects on vascular responses may not necessarily translate into enhanced tissue regeneration [49].

Microgravity is another significant factor affecting the cardiovascular system, which is highly sensitive to changes in gravity. Microgravity induces a cephalad fluid shift and unloads the cardiovascular system from gravitational stress, thereby initiating a deconditioning phenotype reminiscent of aging [34]. In 2003, Gillette-Ferguson et al. first used a GFP transgenic *zebrafish* as a model to investigate the effect of SMG mediated by a NASA-developed bioreactor on the development of *zebrafish* embryos. They found that GFP fluorescence intensity in the heart exhibited the most significant increase in the whole embryo, indicating that the heart may be more sensitive to SMG than other organs of the *zebrafish* embryo [50]. This result was further verified by Shimada et al. [51]. As one of the important indexes reflecting heart function, heart rate is also sensitive to microgravity. Li et al. reported that the microgravity environment simulated by acoustic levitation induced an increase in heart rate in *zebrafish* embryos [52]. This phenomenon was also observed in *zebrafish* embryos after horizontal rotary culture [53]. Furthermore, Sun et al. found that horizontal rotary culture resulted in downregulation of vascular-related genes, such as flk1, flt4, and ephrinB2, in *zebrafish* embryos [53]. These results indicate that SMG may also influence vascular development in *zebrafish*. In a recent study, SMG was found to induce remodeling of the caudal vein plexus network by enhancing angiogenesis in *zebrafish* embryos. Mechanistic studies showed that SMG induced abnormal development of the cardiovascular system through the PI3K-nos2b signaling pathway [54].

**Figure 2:**
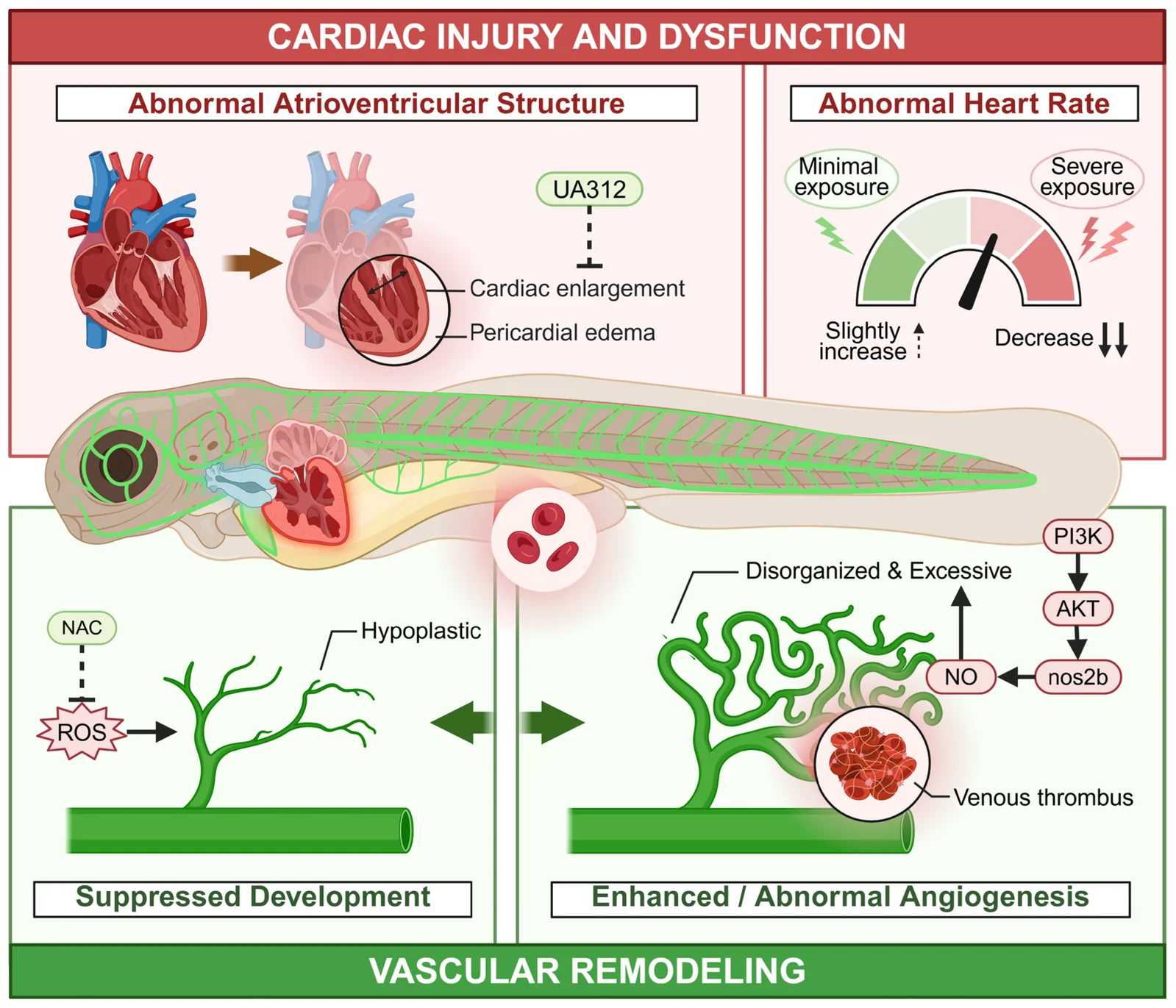
Effect of space environment on cardiovascular system. Created in BioRender. Er, T. (2026) https://BioRender.com/ljrlpjo (accessed on 19 October 2026).

Overall, these studies indicate that radiation and SMG could alter cardiac activity and vascular development in *zebrafish* (Figure 2). However, most available evidence comes from embryos or larvae exposed to short-term ground-based simulations and primarily reflects developmental or vascular changes. In particular, *zebrafish* studies have not yet directly assessed human-relevant outcomes such as orthostatic intolerance, reduced cardiac performance, or post-exposure cardiovascular recovery. Therefore, *zebrafish* offer a valuable model for investigating how spaceflight-related stressors affect cardiovascular development and vascular remodeling, while the relevance of these findings to astronaut cardiovascular deconditioning requires further validation.

### 3.2. Nervous System

The central nervous system (CNS) is a primary research focus in space biology owing to its vital role in physiological and behavioral regulation [4,55]. To investigate these effects, *zebrafish* have been widely used as a model for radiation-induced neurotoxicity, with CNS damage as the most extensively examined endpoint [56]. Doses of 0.1 and 1 Gy of γ-irradiation led to abnormal telencephalic development and neuronal apoptosis in this brain region [25]. Zhao et al. reported that even an extremely low dose of 15 mGy γ-irradiation induced brain cell apoptosis and organelle damage in *zebrafish* embryos, potentially through dysregulation of the Wnt signaling pathway [57]. Structural abnormalities of the nervous system generally reflect underlying functional impairments, which can be characterized by behavioral changes. For example, Houdigui et al. found that prolonged low-dose-rate γ-irradiation (0.5, 5, and 50 mGy/h) altered behavioral responses in the light-dark test in *zebrafish* larvae [56]. Similarly, He et al. reported that low-dose-rate γ-irradiation (0.1, 0.2, and 0.4 mGy/h) induced dose-dependent behavioral responses in *zebrafish* embryos: increased swimming speed and morphological alterations were observed at 0.2 and 0.4 mGy/h, whereas a opposite response was noted at 0.1 mGy/h [58]. In addition to affecting locomotor patterns, low-dose γ-irradiation concurrently disrupted circadian rhythms by altering melatonin secretion, as evidenced by dysregulation of the core clock genes CLOCK1A and BMAL1B [25]. Radiation-induced neurobehavioral deficits are not limited to early development; adult *zebrafish* also exhibit pronounced functional impairments following exposure. Liao et al. reported that radiation exposure increased ROS generation and contributed to DNA damage and cognitive behavioral abnormalities in *zebrafish* [59]. In an adult *zebrafish* cranial-irradiation model, radiation-induced brain injury was associated with increased oxidative stress and inflammatory responses, whereas honokiol exerted protective effects [60].

These behavioral phenotypes point to specific underlying molecular regulatory mechanisms. Xu et al. investigated that significant downregulation of multiple neurotransmitters alongside decreased expression of key neurodevelopmental genes [43]. miRNAs have been reported to play an important role in the development of the *zebrafish* nervous system [61,62]. Furthermore, higher dose rates (0.2 and 0.4 mGy/h) of γ-irradiation upregulated development-associated miRNAs (dre-miR-196a-5p, dre-miR-210-3p, and dre-miR-338), whereas the lowest rate (0.1 mGy/h) suppressed their expression [58]. Except for the CNS, radiation can also influence the peripheral nervous system in *zebrafish*. It was reported that radiation inhibited cell regeneration in *zebrafish* lateral line neuromasts, a sensory system for detecting water motion and pressure gradients, by disrupting Wnt signaling [63,64].

**Figure 3:**
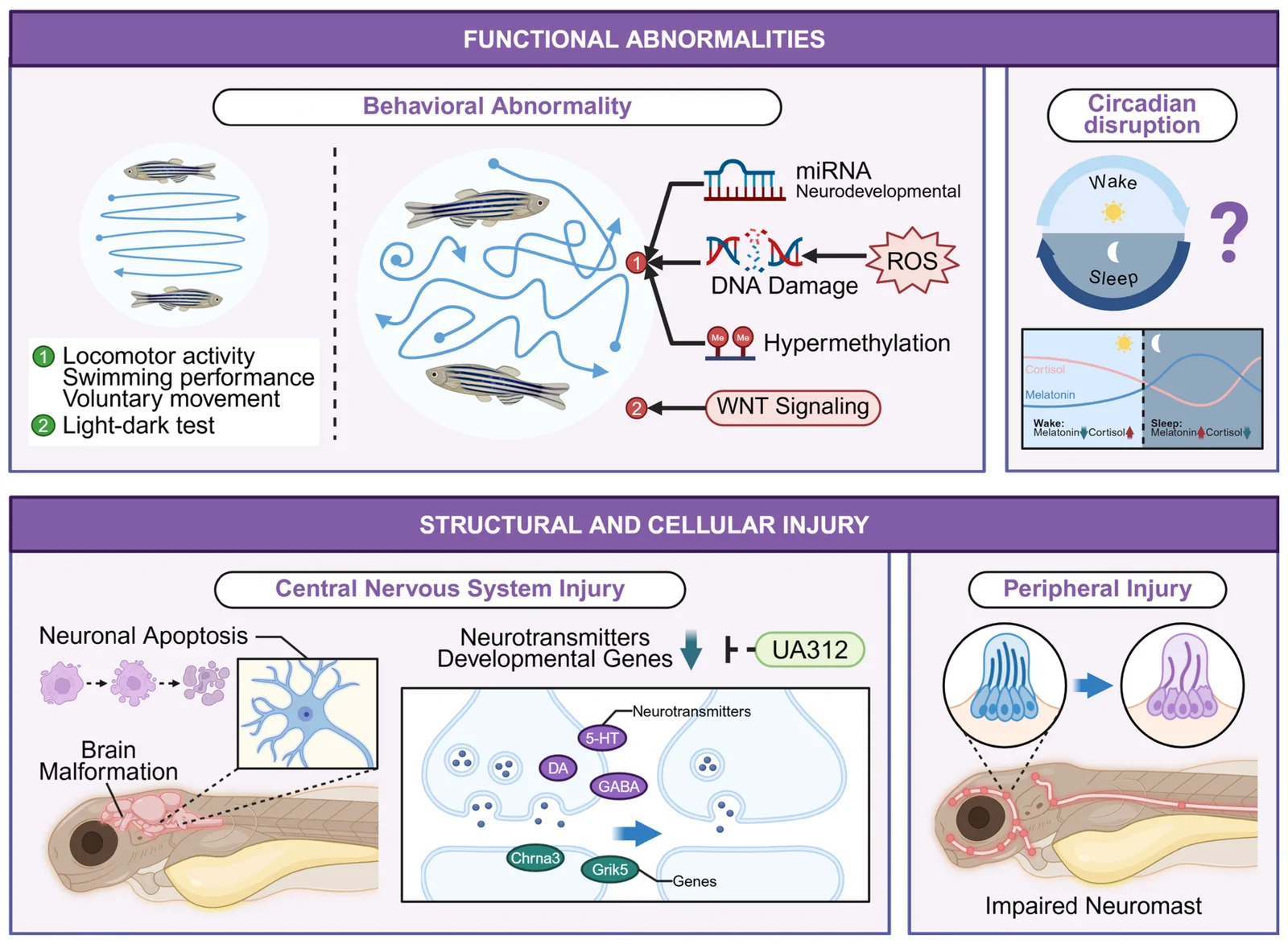
Effect of space environment on nervous system. Created in BioRender. Er, T. (2026) https://BioRender.com/ljrlpjo (accessed on 19 October 2026).

The effects of microgravity on the development of the nervous system have been investigated in mice or even humans [65,66,67]. However, only a few studies have examined the effects of microgravity on *zebrafish* neurodevelopment. For example, Li et al. examined the development and nervous system function of *zebrafish* embryo brains exposed to a microgravity environment simulated by acoustic levitation. They found that embryos exhibited a vague brain-beam profile and abnormal brain morphology under microgravity. Moreover, as an indicator of nervous system function, the voluntary movement dependence of the embryo was increased in response to microgravity stimuli [52]. In addition to the brain, neurons in other organs can also be influenced by microgravity. Shimada et al. demonstrated that microgravity altered gene expression in Rohon-Beard neurons of *zebrafish* embryos [51]. Hypergravity is another factor influencing the health of astronauts during space missions [68]. In a recent study, Salazar et al. reported that hypergravity significantly reduced larval movement frequency and impaired normal swimming behavior, possibly associated with genome-wide hypermethylation [69]. Figure 3 summarizes the reported structural, cellular, and functional changes in the zebrafish nervous system. Thus, *zebrafish* studies provide a valuable complementary model for linking molecular changes to functional outcomes and identifying pathways that may be relevant to spaceflight-associated neurological responses. Since many studies have been conducted in embryos or larvae under simulated conditions, they may provide mechanistic evidence to guide further validation in mammalian models and human spaceflight studies.

### 3.3. Musculoskeletal System

Multiple space environment factors, particularly ionizing radiation, microgravity, and hypergravity, can induce structural alterations in the musculoskeletal system [39,52,69,70]. Commonly observed manifestations include spinal curvature, tail malformation, and overall body deformation, which can all be mitigated by treatment with Phenformin or Metformin [39,52,70]. Among these factors, microgravity conditions have a particularly significant effect on bone development. Simulation of microgravity using a rotating-wall vessel resulted in increased variation in caudal vertebrae number, ranging from 13 to 17, compared with the normal range of 15 to 16 [71]. Carvajal-Agudelo et al. reported that 24 h of 3D-SMG generated using a random positioning machine reduced ossification in multiple developing skeletal elements, potentially due to enhanced bone resorption [72]. Ex vivo-cultured scales from Adult *zebrafish* scales have been used as a model of SMG-induced bone remodeling. Using this model, the same group attributed SMG-associated osteogenic impairment to increased osteoclast activity and decreased osteoblast activity [73]. In an ex vivo model of regenerating *zebrafish* scales exposed to SMG, Kabir et al. reported that an antioxidant-loaded zein nanocage formulation increased osteogenic gene expression, calcification, and the calcium-to-phosphorus ratio, consistent with enhanced scale regeneration [74]. In addition, 2D-SMG exhibited similar structural effects on skeleton development. Edsall et al. exposed *zebrafish* embryos to 2D-SMG using a rotating-wall vessel. At 10 dpf, ossification of the notochord, ceratobranchial 5, opercle, and entopterygoids was delayed, and pharyngeal arch morphology was altered. No corresponding morphological differences were apparent at 35 dpf, whereas adult fish (120 dpf) showed abnormalities in some cranial bones, suggesting both transient and delayed skeletal effects of embryonic SMG exposure [75]. The short- and long-term effects of SMG on *zebrafish* and its molecular mechanisms still need to be further elucidated. Complementary, actual spaceflight experiments in a related teleost model have also demonstrated musculoskeletal responses. Medaka reared aboard the International Space Station showed reduced mineral density in the pharyngeal bone and tooth region, increased osteoclast activity, and early changes in osteoblast- and osteoclast-related gene expression after exposure to microgravity [76,77]. Although these studies were conducted in medaka, these findings provide an in-orbit reference for interpreting ground-based *zebrafish* SMG studies and highlight the need to validate simulated-microgravity findings under actual spaceflight conditions.

Direct musculoskeletal effects of hypergravity have been examined in a limited number of *zebrafish* studies. Aceto et al. reported increased head size and formation of selected cranial bones after 3 G exposure from 5 to 9 dpf, accompanied by differential expression of genes involved in skeletal and muscular system development and function [78]. In a separate study, exposure to 3 or 6 G from 3 to 5 dpf for 48 h did not cause gross changes in cartilage or muscle morphology, but altered cartilage material properties, chondrocyte morphology, extracellular matrix organization, and lower-jaw strain distribution [79]. Taken together, these findings indicate that hypergravity can affect skeletal development and cartilage biomechanics, with the magnitude and nature of the response depending on developmental stage and exposure duration.

**Figure 4:**
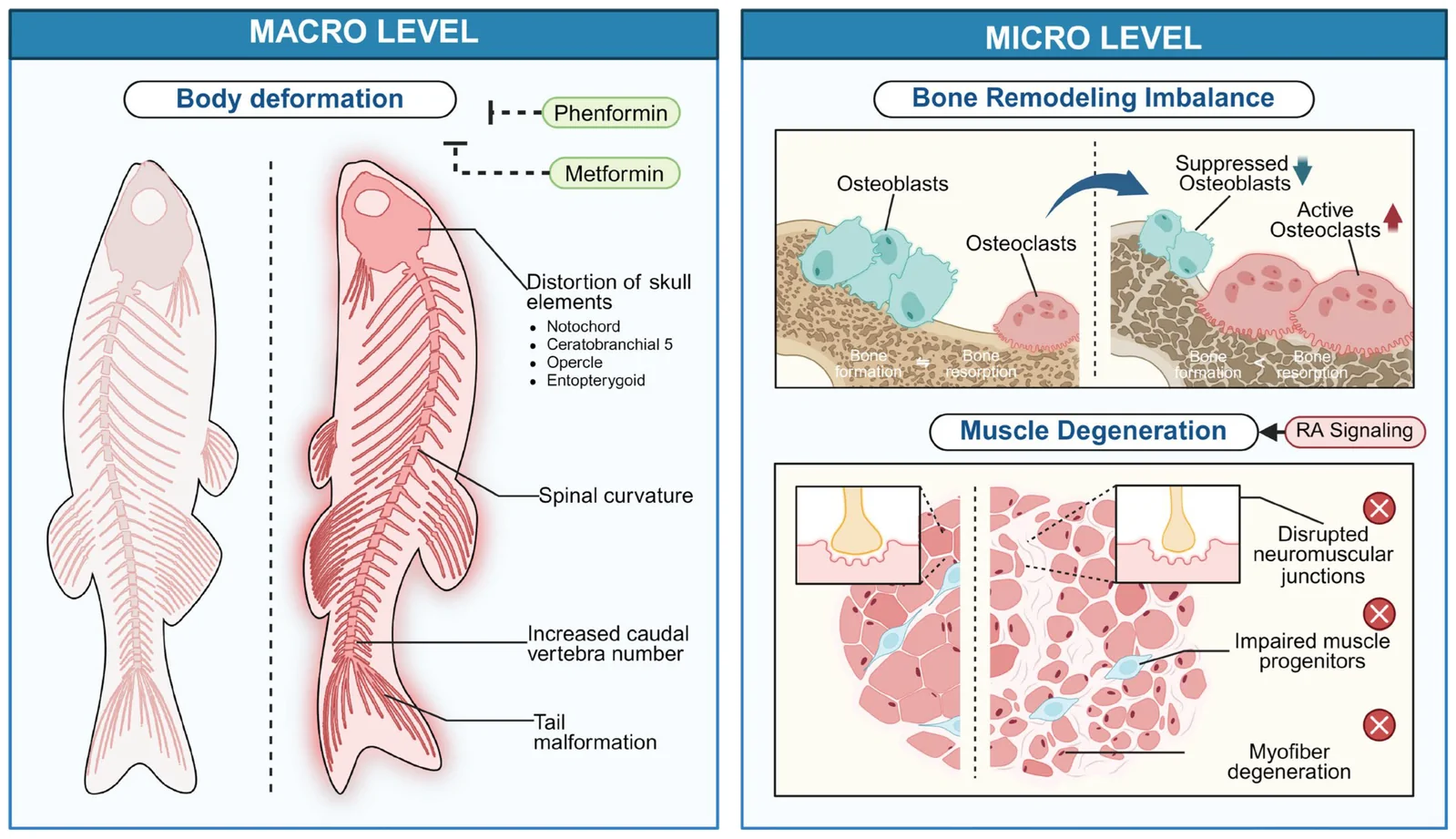
Effect of space environment on musculoskeletal system. Created in BioRender. Er, T. (2026) https://BioRender.com/ljrlpjo (accessed on 19 October 2026).

Skeletal muscle atrophy is the most extensively studied adverse effect of microgravity, and has a significant impact on the muscular function of astronauts [80]. Many studies have investigated the effects of microgravity on muscle and its protective mechanisms in mice [81,82]. However, only a few studies have used *zebrafish* as a model. In 2014, the Japan Aerospace Exploration Agency (JAXA) employed *zebrafish* as a model to investigate the effects of microgravity on muscle via Aquatic Habitat, an aquarium placed in a microgravity environment of the International Space Station (ISS) [83]. In this mission, the researchers detected deterioration of *zebrafish* muscle and investigated whether it recovered upon their return to Earth. Furthermore, Houdigui et al. conducted a ground-based simulation of ionizing radiation exposure and demonstrated that γ-irradiation perturbed key muscle development pathways. These effects manifested as myofiber disorganization, structural alterations in neuromuscular junctions, and impaired muscle progenitor cells. Mechanistically, these phenotypes were linked to dysregulated retinoic acid (RA) signaling, though the precise causal pathways and downstream effectors remain to be fully elucidated [56]. Figure 4 summarizes the reported changes in body and skeletal morphology, bone remodeling, and muscle structure. In summary, the spaceflight environment induces profound musculoskeletal deterioration, including structural bone loss and muscle atrophy. Understanding these changes is critical for developing effective countermeasures to protect astronaut health and ensure mission success during long-duration space exploration.

### 3.4. Sensory System

The ocular system enables visual perception, a process highly vulnerable to the complex space environment [84]. Studies have reported that astronauts on long-duration missions (>1 month of spaceflight) exhibit changes in both visual acuity (due to hyperopic shifts) and ocular structure, including optic disc edema, choroidal folds, and globe flattening. Collectively, these manifestations are termed Spaceflight-Associated Neuro-Ocular Syndrome (SANS), which remains one of the highest-priority health risks in human spaceflight programs [85]. In *zebrafish* embryos, Zhou et al. found that high-LET radiation from ^12^C^6+^ ion beams (2 and 4 Gy) induced significant upregulation of proapoptotic genes, leading to increased ocular apoptosis. Moreover, high-dose (8 Gy) radiation also caused microphthalmia, a vision-related developmental defect. The results of histological analysis showed that ^12^C^6+^ radiation caused a heterogeneous distribution of cells in the ganglion cell layer and inner nuclear cell layer of the embryo eye. These ocular developmental impairments are mechanistically linked to radiation-triggered apoptosis, potentially mediated through a ROS-independent p53 signaling pathway [86]. In addition to high-LET radiation, low-LET radiation, such as γ-ray and X-ray, has also been reported to influence the development of the *zebrafish* embryo eye. Geiger et al. reported that γ-rays at a dose of 10 Gy induced multiple pathological alterations, along with increased apoptosis in the eye of the embryo [87]. Similarly, high doses of X-rays were reported to induce pathological changes and decreased whole-eye diameters in *zebrafish* [88]. Taken together, these studies suggested that both high-LET and low-LET radiation, especially at high doses, can induce ocular damage and disrupt the normal development of the eye in *zebrafish* embryos. *Zebrafish* have also been used to investigate the effect of microgravity on ocular development. Using an HSP70-GFP transgenic line with lens-specific expression, Shimada et al. first demonstrated that both microgravity and hypergravity disrupted lens morphogenesis [89].

The vestibular system is equally critical, which mediates balance and spatial orientation [90]. This system comprises the semicircular canals and otoliths, which function as sensors for angular and linear acceleration, respectively [91]. Spaceflight-induced microgravity results in transient vestibular dysfunction in astronauts, manifesting as impaired balance, gaze control, and motion perception [92]. The vestibulo-ocular reflex (VOR) serves as a key metric for vestibular function [93,94]. As a compensatory movement of the eye, VOR stabilizes retinal images by compensating for head movements induced by gravity. Moorman et al. demonstrated that SMG impairs VOR, with the 30–66 hpf period identified as critical for vestibular development and highly sensitive to gravitational alterations [95]. As the major sensor of linear acceleration and gravity, otoliths have been reported to be involved in SMG-induced abnormal behaviors in *zebrafish*. Moorman et al. found that SMG-exposed larvae exhibited aberrant swimming patterns, including corkscrew motions and vertical loops, accompanied by reduced saccular otolith size or complete otolith loss. The results indicated a significant delay in otolith development, which may contribute to vestibular dysfunction [96]. Mo et al. demonstrated that otoliths are essential for the VOR, as mutant larvae lacking anterior otoliths showed no eye movement response to rotation [97]. However, a recent study reported that delayed otolith development did not have a negative effect on the vestibular circuit development [98]. Hence, the mechanisms of SMG-induced abnormal vestibular development require further investigation.

### 3.5. Reproductive System

Spaceflight adversely affects reproductive function, leading to suppressed testosterone levels and impaired spermatogenesis in males, as well as disrupted estrous cycles in females [99,100,101,102,103]. Reproductive end point was critical for evaluating the function of the reproductive system. Given the characteristics of embryos, medaka fish have already been used to investigate the effects of spaceflight on reproductive endpoints [104]. Hurem et al. demonstrated that subchronic γ-irradiation caused progressive reproductive impairment in females, with embryo production declining within one month and progressing to gonadal regression and sterility after one year, while testicular morphology remained unaffected at equivalent doses [105]. However, a subsequent study using acute radiation exposure confirmed that both X-ray and carbon ion irradiation reduced testis weight and induced testicular cell apoptosis, identifying the mitochondrial apoptotic pathway as a key mechanism underlying this effect [106]. Zhu et al. further elucidated the sex-specific responses to X-ray radiation: male gonads showed extensive disruption, while females exhibited coordinated protective responses [107]. In contrast to these direct parental effects, Guirandy et al. reported that irradiated adults, while exhibiting no immediate reproductive performance deficits, produced non-irradiated progeny with developmental abnormalities, indicating transgenerational genetic instability [108].The increased developmental abnormalities were also shown to be specifically associated with paternal radiation exposure, which impaired sperm morphology and motility [107]. Furthermore, γ-irradiation could also induce masculinization of offspring, associated with increased DNA methylation of key female sex-differentiation genes in ovarian tissue [109]. Collectively, these studies highlight that radiation effects on reproduction depend critically on dose, sex, and the time window of exposure, encompassing both direct organ damage and transgenerational consequences.

### 3.6. Other System

During the Apollo missions, over half of the astronauts exhibited immune suppression and bacterial infections [110]. Subsequent research, including the NASA Twin Study, has consistently shown that spaceflight induces alterations in multiple immune-related pathways [3]. SMG is known to affect both innate and adaptive immunity by impairing immune cell development and proliferation [111,112,113]. Zhu et al. reported that SMG exacerbated the toxicity of viral infection in *zebrafish* embryos by suppressing innate immunity. RNA-seq (GSE145967, corresponding to NASA GeneLab accession GLDS-373) analysis revealed that SMG downregulated genes involved in inflammatory and innate immune responses, including components of the NF-κB, RIG-I-like receptor (RLR), and Toll-like receptor (TLR) signaling pathways. Further investigation demonstrated that SMG inhibited TRIM25, an E3 ubiquitin ligase, thereby impairing RLR pathway activation and disrupting the positive feedback loop essential for antiviral defense [114]. Radiation exerts dual immunomodulatory effects: high doses (>1 Gy) suppress immunity and enhance inflammation, whereas intermediate doses (0.1–1 Gy) mitigate inflammatory responses [115,116]. Traver et al. found that lethal doses of γ-irradiation (20 and 40 Gy) caused specific ablation of hematolymphoid cells and death in adult *zebrafish* [117]. Other studies also reported that γ-irradiation induced oxidative damage in immune cells and *zebrafish* embryos, which could be mitigated by natural antioxidants [118,119]. However, unlike the acute high-dose radiation typically studied in terrestrial models, the space environment presents a scenario of chronic, low-dose radiation exposure. This sustained exposure profile may induce a pro-inflammatory state, potentially elevating long-term risks for chronic degenerative diseases.

The digestive system of *zebrafish*, as in other vertebrates, is derived from the endoderm, including the liver, gallbladder, pancreas and four intestinal domains (intestinal bulb, mid-intestine, hindgut, and proctodeum). Notably, *zebrafish* are stomachless, while the intestinal bulb is considered their functional analog [120]. Beyond its primary roles in nutrient digestion and absorption, the digestive tract also serves as a critical barrier against environmental toxins [121]. Using two complementary low-dose γ-irradiation models, Cahill et al. characterized radiation-associated responses in the *zebrafish* digestive system. In a hypothermic liver model, a very low dose of γ-irradiation (16.34 cGy) altered transcriptomic pathways related to lipid metabolism, wound healing, immune responses, and fibrosis [122]. In a separate torpor-like model, low-dose irradiation induced DNA damage and oxidative stress and altered steroid-signaling, metabolic, and cell-cycle-related pathways in the gastrointestinal tract; importantly, the torpor-like state reduced radiation-associated DNA damage [123]. At higher doses, Wu et al. demonstrated that 10 Gy of γ-irradiation induced hepatic fibrosis and hepatocyte apoptosis in adult *zebrafish*, and that Oligo-Fucoidan (OF) showed protective effects against such damage [124]. Radiation also severely impacted intestinal morphology and function. Jensen et al. demonstrated that a dose of 2 Gy triggered a pathological progression from initial hypertrophy to subsequent atrophy in the intestine, ultimately impairing its function [125]. Furthermore, Park et al. reported that high-dose (45–50 Gy) photon irradiation severely deformed intestinal villi, with mistletoe extract demonstrating protective effects [126]. Thus, these radiation-induced hepatointestinal injuries in *zebrafish* may provide complementary evidence relevant to hepatic and intestinal risks in space.

**Table 1:** Effects on Major Physiological Systems. For in vivo studies, the model indicates the developmental stage at exposure; ex vivo entries identify the isolated tissue used. Embryo (0–72 hpf), Larva (72 hpf–30 dpf), Adult (>90 dpf) [127]. A linear energy transfer (LET) of approximately 10 keV/μm is commonly used as the demarcation between low- and high-LET radiation [128]. * Damage pathway, # Rescue measure.

System	Factor	Model	Effect	Study	Exposure Condition
Cardiovascular system	High-LET	Embryo	Decreased heart rate	[36,37,38]	^56^Fe ion (2, 4 Gy); ^12^C ion (2 Gy); proton (8 MeV/μm; 3, 6, 9, 12, 15 Gy)
		Embryo	Pericardial edema	[36,37]	^56^Fe ion (0.5, 1, 2, 4 Gy); ^12^C ion (2 Gy)
	Low-LET	Embryo	Pericardial edema	[39,42]	X-ray (4 Gy); γ-ray (0.01, 0.05, 0.1, 0.5, 1 Gy)
		Embryo	Decreased heart rate	[39]	X-ray (4 Gy)
		Embryo-Larva	Altered heart rate (0.01~2 Gy stimulation; 10 Gy suppression)	[40,41]	γ-ray (0.01, 0.05, 0.1, 1, 2, 10 Gy)
		Embryo-Larva	Abnormal cardiac structures Tail venous thrombus # UA312	[43]	γ-ray (6 Gy)
		Embryo Adult	Accelerated vessel sprouting Increased inter-ray vessel density	[46]	X-ray (0.5 Gy)
		Adult	Increased angiogenesis and inter-ray vessel density	[49]	LDIR (0.5 Gy)
	LET not specified	Embryo	Suppressed vascular development	[45]	Proton (35 MeV, SOBP; 1, 2, 5 Gy)
	Microgravity	Embryo	Increased heart rate	[52,53]	Acoustic levitation; horizontal rotary culture
		Embryo	Suppressed vascular development	[53]	Horizontal rotary culture
		Embryo	Enhanced angiogenesis	[54]	Bioreactor (MG-IIA, CHN)
Nervous system	Low-LET	Embryo	Neuronal apoptosis and abnormal neuronal development Disrupted circadian rhythm	[25]	γ-ray (0.1, 1 Gy)
		Embryo	Neuronal apoptosis	[57]	γ-ray (15 mGy)
		Embryo-Larva	Altered light-dark behavior * Wnt signaling pathway	[56]	γ-ray (0.5, 5, 50 mGy/h; 0–120 hpf)
		Embryo	Altered swimming speed and neurodevelopmental miRNAs (10.8 mGy stimulation; 21.6, 43.2 mGy suppression)	[58]	γ-ray (0.1, 0.2, 0.4 mGy/h; 12–120 hpf)
		Adult	Abnormal cognitive behaviors * DNA damage via oxidative stress # Honokiol	[59,60]	X-ray (20 Gy)
		Embryo-Larva	Decreased neurotransmitter levels. # UA312	[43]	γ-ray (6 Gy)
		Larva	Inhibited lateral line neuromast regeneration. * Wnt signaling pathway	[64]	X-ray (20, 30 Gy)
	Microgravity	Embryo	Abnormal brain morphology and behavior	[52]	Acoustic levitation
		Embryo	Altered neuronal gene expression	[51]	Bioreactor (NASA)
	Hypergravity	Larva	Behavioral abnormalities * Hypermethylation	[69]	Centrifuge (3 G)
Musculoskeletal system	Low-LET	Embryo	Body deformation (Spinal curvature, tail malformation, and overall body deformation) # Phenformin, Metformin	[39,70]	X-ray (4 Gy); γ-ray (5.2, 5.4 Gy)
		Embryo-Larva	Abnormal muscle development (myofiber, neuromuscular junction, progenitor) * RA signaling pathway	[56]	γ-ray (0.5, 5, 50 mGy/h; 0–120 hpf)
	Microgravity	Embryo	Body deformation	[52]	Acoustic standing wave field
		Embryo-Larva	Increased caudal vertebra number	[71]	Rotating wall vessel
		Larva	Reduced ossification	[72]	Random positioning machine
		Ex vivo-cultured scale	Increased osteoclast activity and decreased osteoblast activity) # Antioxidant protein nanocage	[73,74]	Random positioning machine
		Embryo-Adult	Delayed ossification of skeletal elements (notochord, ceratobranchial 5, opercle, and entopterygoid) and cranial bone abnormalities in adults	[75]	Rotating Wall Vessel
	Hypergravity	Larva	Broadened skull	[78]	Centrifuge (3 G)
		Larva	Altered jaw cartilage	[79]	Centrifuge (3, 6 G)
		Larva	Body deformation	[69]	Centrifuge (3 G)
	Spaceflight	Adult	Muscle deterioration	[83]	ISS
Sensory system	High-LET	Embryo	Microphthalmia (8 Gy) Ocular apoptosis and developmental disruption (ganglion/inner nuclear layer) * ROS-independent p53 signaling pathway	[86]	^12^C^6+^ ion (2, 4, 8 Gy)
	Low-LET	Embryo	Ocular apoptosis Pathological alteration (hypocellularity, layer disorganization, lens opacification)	[87]	γ-ray (10 Gy)
		Embryo	Reduced eye diameter * Oxidative stress	[88]	X-ray (1, 2, 4, 8 Gy)
	Microgravity	Embryo-Larva	Impaired VOR	[95]	Bioreactor (NASA)
		Embryo-Larva	Delayed otolith development (small/missing otolith)	[96]	Bioreactor (NASA)
	Microgravity & Hypergravity	Embryo-Larva	Abnormal lens development	[89]	Bioreactor (NASA); centrifuge (2, 3 G)
Reproductive system	Low & High-LET	Adult	Reduced testis weight Spermatogenic cell apoptosis * Mitochondrial apoptotic pathway	[106]	X-ray (1, 2, 4, 6, 8 Gy); carbon ion (1. 4 gy)
	Low-LET	Adult	Declined embryo production Gonadal regression and damage	[105]	γ-ray (8.7, 53 mGy/h; 27 d)
		Adult	Gonad sexually dimorphic effect (male disruption, female protection) Sperm impairment Progeny developmental defect	[107]	X-ray (1, 5, 10 Gy)
		Adult	Progeny developmental defect	[108]	γ-ray (50 mGy/h; 10 d)
		Adult	Offspring masculinization * DNA methylation of ovarian female sex-differentiation genes	[109]	γ-ray (5 mGy/h; 30 d)
Other system	Low-LET	Adult	Hematolymphoid cell ablation	[117]	γ-ray (20, 40 Gy)
		Embryo	Oxidative damage # Taurine	[118,119]	γ-ray (2, 30 Gy)
		Adult	Hepatic immune dysregulation	[122]	γ-ray (16.34 cGy)
		Adult	DNA damage; oxidative stress; altered metabolism and steroid signaling	[123]	γ-ray (16.34 cGy)
		Adult	Hepatic fibrosis and apoptosis	[124]	γ-ray (10 Gy)
		Adult	Intestinal villi deformation # mistletoe extract	[126]	Photon (45–50 Gy)
	Ionizing radiation	/	Intestinal pathological alteration (hypertrophy, atrophy, functional impairment)	[125]	(2 Gy)
	Microgravity	Embryo	Suppressed innate immunity	[114]	Rotary cell culture system (NASA)

## 4. Future Perspectives and Limitation

The announcement of long-term spaceflight missions, such as those to Mars, has brought the multidimensional and cumulative health risks associated with the space environment to the forefront of scientific concern. *Zebrafish*, with their unique advantages including evolutionary conservation, optical transparency, high fecundity, and genetic tractability, have emerged as an ideal vertebrate model for ground-based studies, providing an indispensable platform for elucidating the molecular mechanisms and physiological pathways underlying space environment-induced damage. This review synthesizes current evidence on the effects of space-relevant environmental factors on *zebrafish*. Ionizing radiation profoundly affects the cardiovascular, immune, and reproductive systems, with the heart identified as an early and sensitive target. Gravity alterations primarily affect the musculoskeletal system, vestibular apparatus, and swim bladder, with responses varying by developmental stage and exposure conditions. Notably, these responses vary across developmental stages and sexes, indicating the need for further investigation.

Despite these advances, current evidence from *zebrafish* space biology remains constrained by several limitations.

(1) Limited translational relevance of *zebrafish* models. Given that many published studies were performed in embryos or larvae, their findings may not fully reflect the physiological responses expected in adult astronauts. Future work should compare different developmental stages and validate key observations in adult *zebrafish* and complementary vertebrate models, thereby clarifying the specific translational contributions of *zebrafish* to space-health research.
(2) Limitations of ground-based space-environment simulations and combined stressors. Ground-based models do not fully reproduce mission-relevant radiation or gravity conditions. In particular, many studies summarized here use acute, single-exposure X-ray or γ-ray irradiation, whereas deep-space missions involve prolonged, lower-dose-rate exposure to a mixed radiation field composed mainly of protons, together with less abundant but biologically important high-energy, high-charge ions. SMG devices may also generate residual acceleration and altered fluid dynamics. In addition, direct *zebrafish* studies combining radiation and SMG remain limited, so single-stressor findings cannot demonstrate whether their joint effects are additive, synergistic, or antagonistic. Future studies should compare the effects of combined radiation and SMG exposure with those of each stressor alone. Other spaceflight-related stressors, including noise, confinement, and circadian rhythm disruption, may also modify biological responses and warrant consideration.
(3) Limited coverage of biological processes and multisystem responses. *Zebrafish* possess well-established advantages and a strong research foundation for studying tissue regeneration, wound healing, gut microbiota, and host–microbe interactions, owing to their regenerative capacity, optical accessibility, and experimental tractability. However, these biological processes remain insufficiently investigated under spaceflight or simulated space conditions, particularly in adult fish. Moreover, most existing studies focus on individual physiological systems, and the cross-system effects of spaceflight-related stressors remain poorly understood. Future work should directly investigate these processes and their multisystem interactions under integrated space-relevant conditions.
(4) Translating phenotypic observations into molecular mechanisms and protective strategies. The molecular mechanisms of many space-environment-induced phenotypes remain unclear, and evidence for reported protective candidates is still preliminary. These agents have generally been explored in embryonic models under limited dosing conditions and acute irradiation, without sufficient information on pharmacokinetics, adult efficacy, dose dependence, formulation, stability, or tolerability. Future studies should combine *zebrafish* genetic tools with systematic pharmacological evaluation to establish causality and translational potential.

From CEBAS (Germany) to CAES (China), closed aquatic ecosystem platforms have advanced substantially, with the latter demonstrating the in-orbit cultivation and reproduction of *zebrafish* during a 43-day mission. Future studies using *zebrafish* in such systems can advance along several key directions. First, further development of in-orbit live imaging could enable longitudinal monitoring of physiological processes, including cardiovascular function, neuronal activity, and cellular turnover. This approach could provide repeated, in situ observations under actual spaceflight conditions. Second, pre-established CRISPR/Cas9-edited *zebrafish* strains, together with appropriate controls, could be used to test candidate genes involved in DNA-damage responses and gravity-related signaling during spaceflight. Finally, ground-based high-throughput screening could identify candidate countermeasures for subsequent targeted in-orbit evaluation. Such stepwise studies could strengthen the assessment of proposed mechanisms and protective effects under spaceflight conditions.

## 5. Conclusions

Taken together, these initiatives would deepen fundamental understanding of organismal adaptation to space, while providing practical knowledge and tools to safeguard crew health during future long-duration, deep-space missions. This progression reflected an essential shift in space life sciences from observational research toward proactive intervention and precision health preservation. In the future, while the physiological responses of *zebrafish* to the space environment remain to be fully characterized, *zebrafish* are expected to gain growing prominence as a prime model for space biology research.

## Data Availability

No new data were created or analyzed in this study. Data sharing is not applicable to this article.
